# Short-term clinical outcomes of a European training programme for robotic colorectal surgery

**DOI:** 10.1007/s00464-020-08184-1

**Published:** 2020-12-07

**Authors:** Sofoklis Panteleimonitis, Danilo Miskovic, Rachelle Bissett-Amess, Nuno Figueiredo, Matthias Turina, Giuseppe Spinoglio, Richard J. Heald, Amjad Parvaiz, On behalf of the EARCS Collaborative

**Affiliations:** 1 School of Health and Care Professions, University of Portsmouth, St Andrews Court, St Michael’s Road, Portsmouth, PO1 2PR UK; 2 St. Mark’s Hospital, London, UK; 3 Champalimaud Foundation, Av. Brasilia, 1400-038 Lisbon, Portugal; 4 Division of Colorectal Surgery and Proctology, University of Zurich Hospital, Moussonstrasse 2, 8044 Zurich, Switzerland; 5 European Institute of Oncology, via Ripamonti 435, Milan, Italy; 6 Pelican Cancer Foundation, Dinwoodie Dr, Basingstoke, RG24 9NN UK; 7 Poole Hospital NHS Trust, Longfleet road, Poole, BH15 2JB UK

**Keywords:** Robotic colorectal surgery, Robotic rectal surgery, Robotic surgery training, Minimally invasive colorectal surgery, Surgical training

## Abstract

**Background:**

Despite there being a considerable amount of published studies on robotic colorectal surgery (RCS) over the last few years, there is a lack of evidence regarding RCS training pathways. This study examines the short-term clinical outcomes of an international RCS training programme (the European Academy of Robotic Colorectal Surgery—EARCS).

**Methods:**

Consecutive cases from 26 European colorectal units who conducted RCS between 2014 and 2018 were included in this study. The baseline characteristics and short-term outcomes of cases performed by EARCS delegates during training were analysed and compared with cases performed by EARCS graduates and proctors.

**Results:**

Data from 1130 RCS procedures were collected and classified into three cohort groups (323 training, 626 graduates and 181 proctors). The training cases conversion rate was 2.2% and R1 resection rate was 1.5%. The three groups were similar in terms of baseline characteristics with the exception of malignant cases and rectal resections performed. With the exception of operative time, blood loss and hospital stay (training vs. graduate vs. proctor: operative time 302, 265, 255 min, *p* < 0.001; blood loss 50, 50, 30 ml, *p* < 0.001; hospital stay 7, 6, 6 days, *p* = 0.003), all remaining short-term outcomes (conversion, 30-day reoperation, 30-day readmission, 30-day mortality, clinical anastomotic leak, complications, R1 resection and lymph node yield) were comparable between the three groups.

**Conclusions:**

Colorectal surgeons learning how to perform RCS under the EARCS-structured training pathway can safely achieve short-term clinical outcomes comparable to their trainers and overcome the learning process in a way that minimises patient harm.

**Supplementary Information:**

The online version of this article (10.1007/s00464-020-08184-1) contains supplementary material, which is available to authorized users.

Since the first robotic colectomy in 2002 [1], robotic surgery has increasingly gained acceptance in colorectal surgery, which is evident from the growing number of studies published on the subject [2–4] and the increasing number of robotic units acquired worldwide [5]. Robotic platforms are reported to offer advantages such as stable 3D views, tremor filtering and angulated instruments with multiple degrees of freedom which may explain this increasing popularity [6, 7].

Although there is convincing evidence to support the safety and feasibility of robotic colorectal surgery, with short and long-term outcomes similar or possibly even superior to those of laparoscopic surgery [8–17], there is little evidence to show how this new technology may be safely implemented for colorectal surgery. For new technologies and methods of operating to be introduced, appropriate training remains imperative in order to ensure patient safety and improved clinical outcomes [18]. Most specialist surgeons depend on attending short courses or visiting centres of excellence in order to gain competence in a new surgical technique [19]. However, without a structured training pathway, surgeons may run the risk of exposing their patients to an increasing number of complications during the learning process. In the UK, LAPCO [20], a national training programme designed for the safe adoption of laparoscopic colorectal surgery, led to improved clinical outcomes and minimised patient harm, while surgeons were gaining competency in laparoscopic colorectal surgery [19]. Recognising such a need for robotic colorectal surgery, the European Academy of Robotic Colorectal Surgery (EARCS) was founded (https://earcs.pt) under the auspices of the Champalimaud Foundation in Lisbon, Portugal. EARCS provides a framework and guidelines for selecting appropriate surgeons, skill courses and direct supervision of clinical cases for robotic colorectal surgery. EARCS is audited by collecting clinical results, while surgical performance is evaluated using a structured process for formative and summative assessment. Since October 2014, a total of 148 surgeons have registered with the training programme and 76 surgeons from 54 centres in 15 European countries have graduated from the academy [21].

The aim of this study is to examine the short-term outcomes of a structured training programme for robotic colorectal surgery in an international setting. The presented data give an insight into the training experience of robotic colorectal surgeons across Europe and addresses the paucity of evidence in robotic colorectal surgery training methods on the path to attaining independent practice.

## Materials and methods

Consecutive cases from 26 European colorectal units who performed robotic colorectal surgery between 2014 and 2018 were included in this study. Each centre prospectively collected the baseline characteristics and short-term outcomes of all participating patients. The inclusion criteria included all elective patients eligible for robotic colorectal surgery. Non-colorectal cases were excluded.

Cases were either performed by an EARCS proctor, an EARCS graduate or as part of a training case where an EARCS proctor would train an EARCS delegate. A total of 35 surgeons participated in this study with 7 proctors supervising all training cases. The short-term clinical and oncological outcomes of all cases performed as part of a training case were analysed and compared with cases performed by an EARCS graduate or proctor as a control. Additionally, conversion and complication cumulative sum (CUSUM) charts for the first 10 training cases were constructed to identify whether there was a change in outcome trend during training.

Cancer cases followed local pre and post-operative guidelines and were discussed in the institutional multidisciplinary team meeting (MDT) prior to initiating any type of treatment. Neoadjuvant treatment was given according to local guidelines following MDT discussion. Modified enhanced recovery programmes after surgery (ERAS) were used as standard at all colorectal units in this study [22]. The decision on whether to perform the surgeries robotically was based on surgeon discretion as there were no set criteria for robotic surgery allocation. The majority of the participating centres used the robot for rectal cancer cases only. Robotic surgeries were either performed with the da Vinci Xi, da Vinci Si or da Vinci X models. A standardised fully robotic single docking technique was applied for all surgeries as taught in the EARCS programme [23, 24]. With the exception of robotic setup and docking, the operative modules were the same for all robotic systems.

All included patients signed an informed consent form allowing their data to be used for analysis and research. The requirements for anonymisation of personal dataset by the Data Protection Act 1998 were satisfied.

### Training programme

As the programme was designed for established specialist surgeons, it had different characteristics in its structure compared with training schemes for surgical trainees. Expert trainers were identified and appointed to provide training with a national coordinator and a centre for educational assessment and research. As a prerequisite, expert trainers were required to have conducted a minimum of 200 robotic colorectal resections and to submit a double blinded video assessment of their operative technique.

Of note is that laparoscopic surgery experience was not considered as a prerequisite for delegates to be accepted to the programme. Delegates were encouraged to perform at least 30 h of simulation exercises before the training programme commenced, to familiarise themselves with the robotic console. In addition, each surgeon was required to complete the online modules for the robotic system they were going to be trained on.

The final training step involved hands-on training at one of the faculty member’s hospitals, followed by supervised training at the delegate’s own hospital. All cases were performed under the direct supervision of the faculty who provided mentor support. Delegates and proctors both completed a Global Assessment Score (GAS) form after each proctored (training) case [18]. The number of proctored cases required for each delegate varied with the proficiency of the delegate and was decided by the proctors based on the GAS form as a formative assessment. After completing the required number of proctored cases, delegates were expected to take a final assessment by submitting two anonymized and unedited videos of self-performed robotic anterior resections, including splenic flexure mobilisation, for blinded assessment by the EARCS surgical competency assessment committee using the Robotic Colorectal Assessment Tool (RCAT) as a summative assessment.

EARCS did not have a separate programme for colonic and rectal/pelvic dissection. This is because the aim of the programme was to make surgeons competent in both colonic and rectal surgery. As part of the training for rectal surgery, surgeons were assessed on the modules for colonic dissection, including transection of the vascular pedicle, mobilisation of the colon and splenic flexure mobilisation. In terms of the first training cases performed, it was encouraged to start with easier cases, such as sigmoid or rectosigmoid cancers, and then moving onto the lower rectal cancers. However, this was largely dependent on case availability.

### Data collection and outcome assessment

All data were collected from prospectively maintained databases from each institution and sent to a central data manager who collated the data. The baseline characteristics and short-term surgical outcomes of all elective patients receiving robotic colorectal surgery were collected and analysed. Baseline characteristics included age, body mass index (BMI), gender, American Society of Anaesthesiologists (ASA) grade, malignant vs. benign surgery, neoadjuvant treatment, pathological T and N stage, and the type of operation performed. Perioperative data included operative time, estimated blood loss (EBL) and conversion to open surgery (defined as any incision needed to either mobilise the colon or rectum or ligate the vessels). Post-operative clinical data examined included length of stay (LOS), post-operative complications—Clavien–Dindo (CD) classification [25], 30-day reoperation (defined as any operation requiring a general anaesthetic within 30 days from surgery), 30-day readmission, 30-day mortality and clinical anastomotic leak (defined as any anastomotic leak requiring another intervention, such as external drainage or reoperation). Oncological outcomes examined by a pathologist included lymph node yield and circumferential resection margin (CRM) clearance. CD complication grades 1–2 and 3–5 were grouped together for the purposes of statistical analysis.

A subgroup analysis was performed for patients receiving robotic rectal resections in order to investigate whether conclusions similar to those of the complete cohort can be reached when specifically looking at rectal resections; therefore, eliminating any bias arising from differences in the nature of the procedures performed between the three groups.

### Statistical analysis

Data were analysed using IBM SPSS version 24 (SPSS Inc., Chicago, IL, USA). Non-parametric data were expressed as median with interquartile range and parametric data as mean with standard deviation. When investigating all three cohorts, baseline demographic and clinical characteristics were compared using the *χ*^2^ test for categorical variables, Kruskal Wallis test for non-parametric continuous variables and one-way ANOVA for parametric continuous variables. *P* values of <0.05 were considered statistically significant.

Univariate binary logistic regression analysis was performed to assess whether surgeon role (training, graduate, proctor) affected CD 1–2 and CD 3–5 complication rates. Following this, a multivariate model was applied where surgeon role was adjusted for all clinically relevant variables (gender, age, BMI, ASA grade, rectal resection, neoadjuvant treatment, cancer case). For the purpose of binary logistic regression, missing values were replaced with the series mean (11 missing values for ASA, 53 for BMI). The constant was included in the analysis model and data are presented as odds ratio, 95% confidence interval and *p* value.

CUSUM curves for conversion rate and CD 3–5 complications were charted in order to assess the trend of these outcomes during the training process [26, 27]. For the construction of the CUSUM charts the target was the conversion and CD 3–5 complication rate of all graduate cases (*X*_0_). Therefore, the CUSUM score is the cumulative sum of *X*_*i*_ – *X*_0_ where *X*_*i*_ represents the average conversion or CD 3–5 complication rate of each succeeding training case for the first ten cases (i.e., the average of all first training cases, followed by the average of all the second training cases, etc.). In the CUSUM chart the *x*-axis represents the consecutive cases and the *y*-axis the CUSUM score. The CUSUM curves ascend when the set target is not reached, which reflects an ongoing learning process. When the curve plateaus or descends the target is achieved or superseded, representing the end of the learning process. CUSUM curves are only charted for the first 10 cases, since after 10 the number of surgeons performing further training cases significantly drops.

## Results

A total of 1130 patients were included in this study. Of those, 323 (28.6%) were EARCS training cases, 626 (55.4%) were performed by EARCS graduates and 181 (16%) by EARCS proctors.

### Cohort characteristics

The three groups were similar in terms of baseline characteristics. However, the proctor group had a greater proportion of malignant cases and rectal resections. The baseline characteristics of the three groups are summarised in Table 1.

**Table 1:** Baseline characteristics of robotic colorectal procedures

	Training (*n* = 323)	Graduate (*n* = 626)	Proctor (*n* = 181)	*p* value
Age (years)	65 (56–75)	67 (57–74)	66 (59–74)	0.485
BMI (kg/m^2^)	26 (24–29)	26 (23–29)	26 (24–28)	0.497
Gender				
Male	189 (58.5%)	376 (60.1%)	112 (61.9%)	
Female	134 (41.5%)	250 (39.9%)	69 (38.1%)	0.756
ASA grade				
1	46 (14.3%)	75 (12.1%)	25 (14.1%)	0.147
2	202 (62.7%)	408 (65.8%)	127 (71.8%)	
3	74 (23%)	134 (21.6%)	25 (14.1%)	
4	0	3 (0.5%)	0	
Malignant	289 (89.5%)	561 (89.6%)	179 (98.9%)	<*0.001*
Neoadjuvant Tx	43 (26.9%)	118 (29.3%)	58 (34.1%)	0.330
T stage				
0	23 (8.6%)	55 (10.6%)	11 (6.3%)	0.555
1	34 (12.6%)	60 (11.6%)	25 (14.4%)	
2	67 (24.9%)	141 (27.3%)	50 (28.7%)	
3	122 (45.4%)	232 (44.9%)	78 (44.8%)	
4	23 (8.6%)	29 (5.6%)	10 (5.7%)	
N stage				
0	157 (66%)	283 (66.4%)	116 (67.1%)	0.647
1	58 (24.4%)	97 (22.8%)	45 (26%)	
2	23 (9.7%)	46 (10.8%)	12 (6.9%)	
Operations				
Anterior resection	191 (59.1%)	367 (58.6%)	139 (76.8%)	*0.018*
APER	26 (8%)	68 (10.9%)	10 (5.5%)	
Hartman’s	3 (0.9%)	2 (0.3%)	3 (1.7%)	
Right hemicolectomy	47 (14.6%)	83 (13.3%)	10 (5.5%)	
Left hemicolectomy	19 (5.9%)	28 (4.5%)	7 (3.9%)	
Sigmoid resection	24 (7.4%)	48 (7.7%)	9 (5.0%)	
Completion proctectomy	3 (0.9%)	4 (0.6%)	0	
Panprocto- or proctocolectomy	2 (0.6%)	4 (0.6%)	1 (0.6%)	
Rectopexy	7 (2.2%)	15 (2.4%)	1 (0.6%)	
Subtotal colectomy	1 (0.3%)	3 (0.5%)	1 (0.6%)	
Other	0	4 (0.6%)	0	
Rectal resections	225 (69.7%)	448 (71.6%)	153 (84.5%)	*0.001*

Statistically significant values are given in italics

*BMI* body mass index, *ASA* American Society of Anaesthesiologists, *APER* abdominoperineal excision

### Short-term outcomes

The short-term outcomes of the three cohorts are detailed in Table 2. There was a relatively low number of conversions, complications, 30-day mortality and R1 resections (for all malignant cases) observed in the training cases, as well as the graduate and proctor cases.

**Table 2:** Short-term outcomes of robotic colorectal procedures

	Training (*n* = 323)	Graduate (*n* = 626)	Proctor (*n* = 181)	*p* value
Conversion	7 (2.2%)	21 (3.4%)	5 (2.8%)	0.583
Operation time (min)	302 (230–390)	265 (200–353)	255 (202–342)	<*0.001*
EBL (ml)	50 (20–100)	50 (20–100)	30 (10–100)	<*0.001*
LOS (days)	7 (5–10)	6 (4–9)	6 (3–8)	*0.003*
30-day reoperation	21 (6.5%)	39 (6.2%)	10 (5.5%)	0.908
30-day readmission	23 (7.1%)	51 (8.1%)	15 (8.3%)	0.835
30-day mortality	1 (0.3%)	2 (0.3%)	0	0.750
Anastomotic leak	9/286 (3.1%)	17/538 (3.2%)	6/166 (3.3%)	0.954
Complications (Clavien–Dindo)				
I or II	40 (12.4%)	89 (14.2%)	20 (11%)	0.714
III to V	33 (10.2%)	53 (8.5%)	17 (9.4%)	
R1 resection	3/203 (1.5%)	6/355 (1.7%)	3/133 (2.3%)	0.863
Lymph node yield	18 (13–25)	18 (13–25)	18 (13–24)	0.778

Statistically significant values are given in italics

*EBL* estimated blood loss, *LOS* length of stay

The conversion rate, 30-day reoperation rate, 30-day readmission rate, 30-day mortality rate, clinical anastomotic leak rate, post-operative complications, CRM clearance and lymph node yield did not differ significantly between the three groups.

The median operation time was shorter in the proctor group and longer in the training group (training vs. graduate vs. proctor: 302, 265, 255 min; *p* < 0.001). The EBL was lower in the proctor group (training vs. graduate vs. proctor: 50, 50, 30 ml; *p* < 0.001). There was a difference in LOS favouring proctors and graduates, when compared to training cases (training vs. graduate vs. proctor: 7, 6, 6 days; *p* = 0.003).

### Logistic regression analysis for post-operative complications

Univariate logistic regression analysis showed that surgeon role did not affect post-operative CD 1–2 or CD 3–5 complications. This was also the case in the multivariate regression analysis when other clinically relevant factors were adjusted for (gender, age, BMI, ASA grade, rectal resection, neoadjuvant treatment, cancer case). Both univariate and multivariate analysis demonstrated that rectal resection was a risk factor for CD 1–2 and CD 3–5 complications. The logistic regression analysis results for CD 1–2 and CD 3–5 complications are presented in Tables 3 and 4, respectively.

**Table 3:** Univariate and multivariate binary logistic regression for Clavien–Dindo 1–2 complications

	OR	Univariate		*p* value	OR	Multivariate		*p* value
		95% CI lower	95% CI upper			95% CI lower	95% CI upper	
Surgeon role (proctor)				0.477				0.364
Surgeon role (graduate)	0.879	0.497	1.555	0.657	0.782	0.436	1.402	0.409
Surgeon role (training)	1.173	0.786	1.749	0.435	1.136	0.758	1.701	0.537
Gender (male)	1.290	0.900	1.849	0.166	1.180	0.818	1.702	0.376
Age	1.002	0.988	1.016	0.770	0.999	0.984	1.015	0.937
BMI	1.031	0.993	1.070	0.113	1.022	0.984	1.062	0.260
ASA grade	1.277	0.950	1.716	0.105	1.205	0.871	1.667	0.260
Rectal dissection	1.871	1.199	2.920	*0.006*	1.661	1.028	2.685	*0.038*
Neoadjuvant Tx	1.578	1.058	2.352	*0.025*	1.372	0.899	2.094	0.143
Cancer	1.610	0.794	3.266	0.187	1.160	0.539	2.496	0.705

Statistically significant values are given in italics

*OR* odds ratio, *CI* confidence interval, *BMI* body mass index, *ASA* American Society of Anaesthesiologists

**Table 4:** Univariate and multivariate binary logistic regression for Clavien–Dindo 3–5 complications

	OR	Univariate		*p* value	OR	Multivariate		*p* value
		95% CI lower	95% CI upper			95% CI lower	95% CI upper	
Surgeon role (proctor)				0.668				0.668
Surgeon role (graduate)	0.911	0.492	1.686	0.767	0.844	0.448	1.589	0.599
Surgeon role (training)	0.813	0.515	1.284	0.374	0.810	0.510	1.288	0.374
Gender (male)	1.399	0.911	2.148	0.125	1.346	0.870	2.085	0.182
Age	0.987	0.972	1.002	0.099	0.986	0.968	1.004	0.116
BMI	0.997	0.953	1.044	0.899	0.989	0.944	1.035	0.626
ASA grade	1.017	0.718	1.439	0.926	1.108	0.756	1.624	0.599
Rectal dissection	2.737	1.507	4.973	*0.001*	2.807	1.476	5.339	*0.002*
Neoadjuvant Tx	1.296	0.800	2.100	0.292	1.011	0.610	1.675	0.967
Cancer	1.182	0.557	2.509	0.662	0.818	0.351	1.907	0.641

Statistically significant values are given in italics

*OR* odds ratio, *CI* confidence interval, *BMI* body mass index, *ASA* American Society of Anaesthesiologists

### Cumulative sum (CUSUM) charts

**Fig.1:**
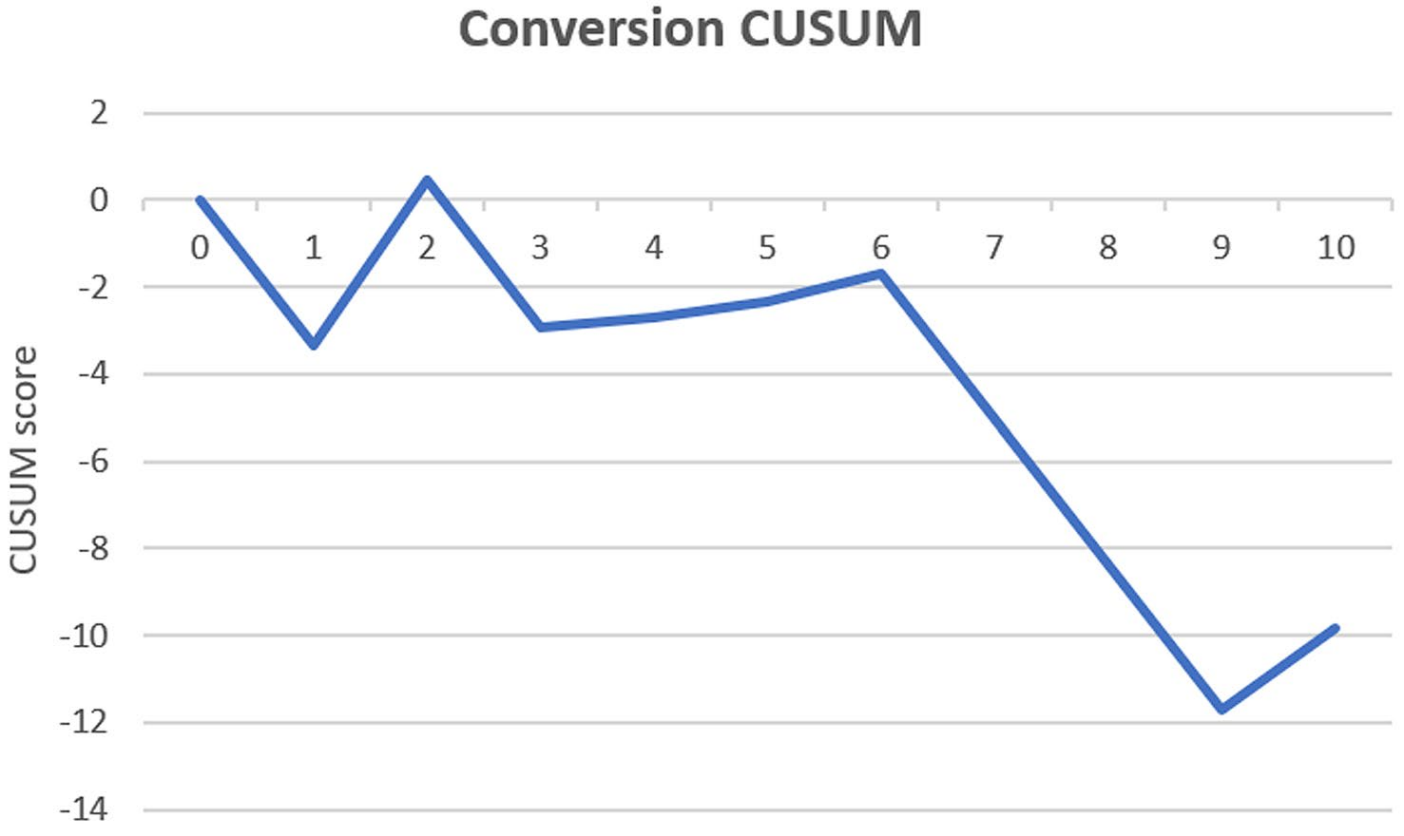
CUSUM conversion

**Fig. 2:**
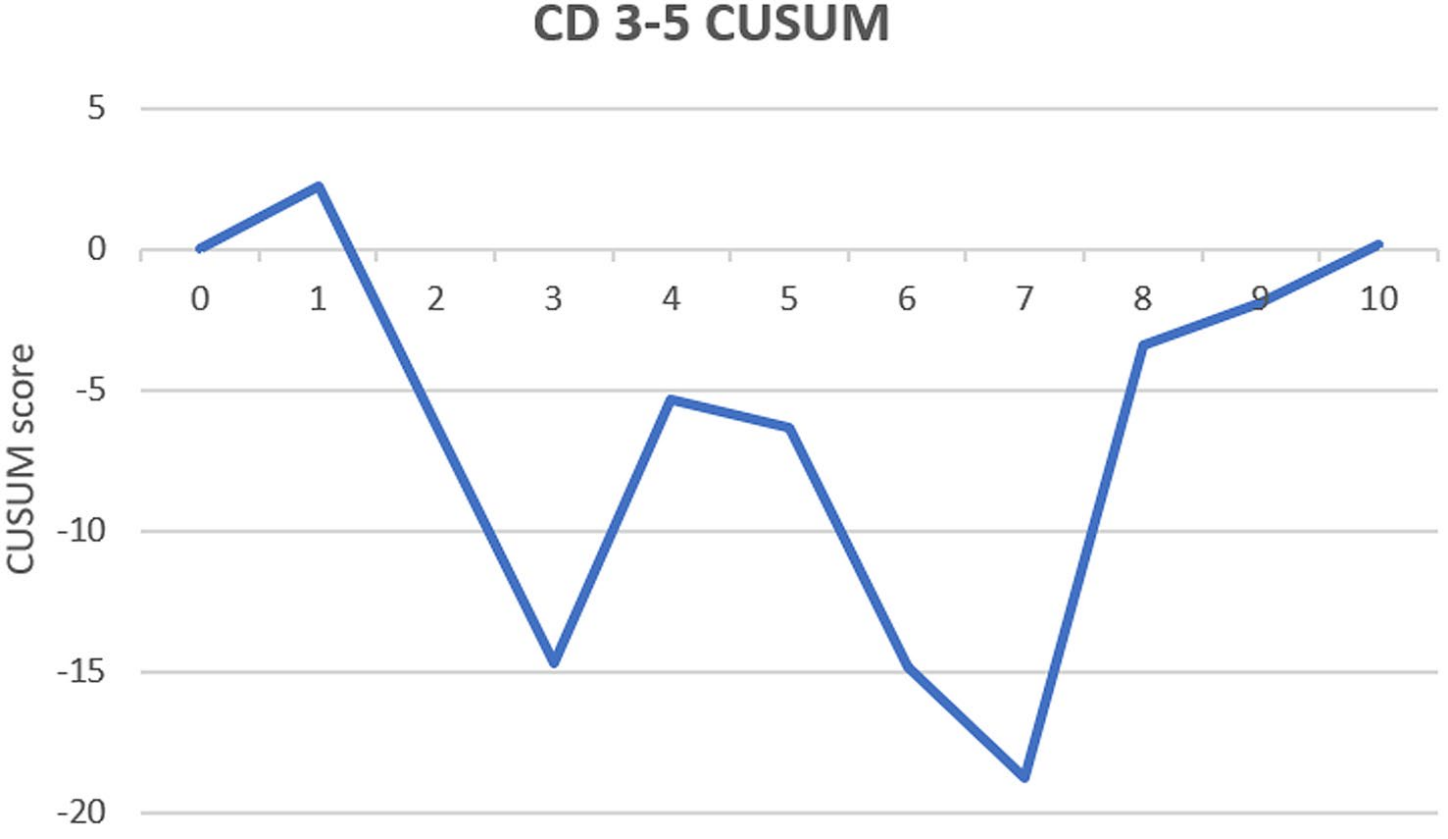
CUSUM CD 3–5

The CUSUM charts for conversion (Fig. 1) and complications CD 3–5 (Fig. 2) did not indicate a positive or negative trend over the first 10 cases, demonstrating that these outcomes are maintained on target during the learning process.

### Subgroups analysis for patients receiving robotic rectal resections

There were 826 patients that had robotic rectal resections. Of these, 225 (27.2%) were training cases, 448 (54.2%) were performed by a graduate and 153 (18.5%) by a proctor. The baseline characteristics and short-term outcomes are summarised in Tables 5 and 6. The three groups were similar in terms of baseline characteristics, procedures performed and short-term outcomes, with the exception of operation time, EBL and LOS. Operation time was longer in the training group (training vs. graduate vs. proctor: 340.5, 300, 260 min; *p* < 0.001), EBL was again lower in the proctor group (training vs. graduate vs. proctor: 50, 50, 32.5 ml; *p* < 0.001) and LOS favoured the proctor and graduate cohorts (training vs. graduate vs. proctor: 7, 6, 6 days; *p* = 0.044). Univariate and multivariate logistic regression analysis again showed that surgeon role did not affect CD 1–2 or CD 3–5 complications.

**Table 5:** Baseline characteristics of robotic rectal resections

	Training (*n* = 225)	Graduate (*n* = 448)	Proctor (*n* = 153)	*p* value
Age (years)	63 (55–74)	66 (57–73)	66 (60–74)	0.109
BMI (kg/m^2^)	26 (24–29)	26 (23–30)	26 (24–28)	0.590
Gender				
Male	141 (62.7%)	275 (61.4%)	101 (66%)	0.593
Female	84 (37.3%)	173 (38.6%)	52 (34%)	
ASA grade				
1	31 (13.8%)	54 (12.2%)	19 (12.8%)	0.077
2	133 (59.4%)	293 (66.1%)	109 (73.2%)	
3	60 (26.8%)	94 (21.2%)	21 (14.1%)	
4	0	2 (0.5%)	0	
Malignant	218 (96.9%)	427 (95.3%)	152 (99.3%)	0.060
Neoadjuvant Tx	43 (41.7%)	115 (39.9%)	58 (40.6%)	0.949
T stage				
0	15 (7.3%)	39 (9.9%)	11 (7.5%)	0.950
1	26 (12.7%)	46 (11.7%)	20 (13.6%)	
2	58 (28.3%)	112 (28.6%)	43 (29.3%)	
3	93 (45.4%)	176 (44.9%)	67 (45.6%)	
4	13 (6.3%)	19 (4.8%)	6 (4.1%)	
N stage				
0	108 (62.4%)	192 (63.8%)	97 (66.4%)	0.185
1	49 (28.3%)	70 (23.3%)	40 (27.4%)	
2	16 (9.2%)	39 (13.0%)	9 (6.2%)	
Operations				
Anterior resection	191 (84.9%)	367 (81.9%)	139 (90.8%)	0.106
APER	26 (11.6%)	68 (15.2%)	10 (6.5%)	
Hartman’s	3 (1.3%)	2 (0.4%)	3 (2%)	
Completion proctectomy	3 (1.3%)	4 (0.9%)	0	
Panprocto- or proctocolectomy	2 (0.9%)	4 (0.9%)	1 (0.7%)	
Other	0	3 (0.7%)	0	

*BMI* body mass index, *ASA* American Society of Anaesthesiologists, *APER* abdominoperineal excision

**Table 6:** Short-term outcomes of robotic rectal resections

	Training (*n* = 225)	Graduate (*n* = 448)	Proctor (*n* = 153)	*p* value
Conversion	6 (2.7%)	18 (4%)	3 (2%)	0.390
Median operation time (min)	340.5 (270–405)	300 (224.5–375)	260 (205–348)	<*0.001*
EBL (ml)	50 (20–100)	50 (20–100)	32.5 (8.75–100)	<*0.001*
LOS (days)	7 (5–11)	6 (4–10)	6 (4–9)	*0.044*
Reoperation	16 (7.1%)	34 (7.6%)	10 (6.5%)	0.905
Readmission	21 (9.3%)	41 (9.2%)	15 (9.8%)	0.972
30-day mortality	1 (0.4%)	1 (0.2%)	0	0.684
Anastomotic leak	7/195 (3.6%)	15/375 (4%)	6/139 (4.3%)	0.943
Complications (Clavien–Dindo)				
I or II	32 (14.2%)	72 (16.1%)	19 (12.4%)	0.795
III to V	27 (12%)	47 (10.5%)	16 (10.5%)	
R1 resection	3/159 (1.9%)	6/272 (2.2%)	3/115 (2.6%)	0.922
Lymph node harvest	18 (13–24)	18 (12–25)	17 (12–23)	0.732

Statistically significant values are given in italics

*EBL* estimated blood loss, *LOS* length of stay

## Discussion

Robotic surgery is greatly increasing in popularity among the colorectal surgical community. Structured mentorship training programmes are essential for the safe adoption of any new surgical technique. While several studies on robotic colorectal surgery have been published over the last few years, there is a lack of evidence regarding robotic colorectal surgery training pathways. In other specialties such as urology, training schemes for robotic surgery have been described, although there is still a recognised need for a structured, standardised curriculum and robust credentialing guidelines for proficiency [28–33]. To our knowledge, in colorectal surgery three studies discuss a training pathway for robotic surgery [18, 34, 35], but only include a relatively small number of surgeons and cases.

This study examines the outcomes of 1130 robotic colorectal cases across 26 centres in Europe and its results suggest that the EARCS programme facilitates safe and effective training of robotic colorectal surgery while ensuring good clinical outcomes. This is demonstrated by examining the short-term surgical outcomes of robotic colorectal surgeries performed by three cohorts: EARCS delegates during training, EARCS graduates and EARCS proctors.

A similar clinical and oncological short-term outcome profile was observed between the three groups, with the exception of operative time, EBL and LOS. This suggests that colorectal surgeons in training, learning how to perform robotic colorectal surgery under the EARCS-structured training pathway, can safely achieve short-term surgical outcomes comparable to those of the EARCS graduates and proctors. This was also confirmed in binary logistic regression analysis where surgeon role (training vs. graduate vs. proctor) was not found to affect CD 1–2 or CD 3–5 complications (see Tables 3 and 4). Additionally, the short-term outcomes of the EARCS graduate and proctor cohorts did not differ significantly, including operation time and length of stay, with the exception of EBL which was lower in the proctor group. The inference that EARCS graduates can achieve short-term outcomes similar to those who trained them is suggestive of the effectiveness of the training programme. Finally, CUSUM graph analysis of the first 10 training cases for conversion rate and CD 3–5 complications, showed no clear positive or negative trend, demonstrating that patient outcomes are stable during the learning process.

The only differences in outcomes observed between the three groups were in operation time, EBL and LOS. Median operation time was shortest in the proctor group and about 47 min shorter than in the training cases. This finding is comparable to outcomes of previous studies for training in laparoscopic colorectal surgery [36–39]. Although EBL was statistically significantly different between the three groups (training vs. graduate vs. proctor: 50, 50, 20 ml; *p* < 0.001), this is probably not clinically relevant and did not translate into a higher rate of post-operative complications.

Interestingly, LOS was different between the three cohorts, with the training cases being discharged one day later than the proctor and graduate cases. There are several factors that could have led to the observed results, in addition to the higher EBL observed in the training groups. Firstly, there were a higher number of benign resections in the graduate and training groups. These cases often include inflammatory conditions such as inflammatory bowel disease (IBD) or diverticulitis, which often take longer to recover from due to associated inflammatory pathology. Secondly, there are a larger number of abdominoperineal excisions (APERs) in the training group compared to the proctor group, which often have a more complex recovery. Thirdly, the difference in LOS could be due to differences in the discharge criteria of the 26 different centres across the 13 different countries, with some institutions behaving more proactively to discharge patients as early as possible due to associated bed pressures. However, it should be noted that the difference in LOS could be due to associated unaccounted surgical morbidity, which if higher in the training cohort could lead to a longer LOS. In two studies published by the Cleveland clinic group [40, 41], it was concluded that LOS, readmission rate and mortality can predict surgical morbidity in colorectal resections. Considering that our mortality and readmission rates are similar between groups, the difference in LOS could be attributed to associated morbidity.

A subgroup analysis of rectal resections yielded similar results to those of the overall cohort. Baseline characteristics, procedures performed and short-term outcomes were similar between the three groups, with the exception of operation time, EBL and LOS. This demonstrates that the conclusions drawn from the results of the overall examined cohort can be applied to robotic rectal resection surgery.

As far as we are aware there are only three previous studies describing robotic colorectal surgery training programmes [18, 34, 35]. The study by Winder et al. [34] offers a comprehensive description of a robotic training programme but includes a mix of general surgical cases, does not state how many colorectal resections were included and does not offer any description of surgical outcomes during the training process. Therefore, the study’s conclusions cannot be considered when discussing robotic colorectal surgery training pathways. The second study by Bell et al. [35] describes the robotic colorectal surgery training pathway of four surgeons in an academic hospital and presents the surgical outcomes of the surgeries performed (*n* = 48) during the training pathway. This study is descriptive and offers no comparison of outcomes for cases performed during or after training. The third study [18] was the first published study to report data from EARCS and included 82 robotic rectal resections performed by three EARCS graduates from two different centres, 30 of which were training cases. Similar to our study, there was no difference in the peri- or post-operative outcomes between the training and graduate groups with the exception of operative time, which was 36 min longer in the training cases.

To our knowledge, the presented study is the largest of its kind and is unique in comparing data between training cases, cases performed by delegates after they had completed their training and cases performed by proctors. Moreover, as far as we are aware this represents the first and largest European robotic colorectal surgery case series investigated to date, with over 1000 cases included from 26 different European centres.

Acknowledging our study limitations, there is obvious selection bias on the process of recruiting patients for proctorship, training and acquiring proficiency. Secondly, the database was derived from surgeon-reported data with its inherit risks of inadequate data entry and not all EARCS graduates submitted their data for this study. Additionally, there are differences in the proportion of benign cases between the three groups and there is a mixture of colorectal procedures included in this study with the proportion of those differing between the three investigated cohorts. However, a subgroup analysis of only rectal resections has demonstrated that the results are similar to those of the overall cohort and there are no baseline characteristic differences between the three groups for rectal resections. Moreover, multivariate regression analysis has mitigated for baseline characteristic differences and demonstrated that surgeon role (training vs. graduate vs. proctor) does not affect complication rates. Considering further limitations, although the large number of centres participating in this study greatly increases the external validity of the investigated outcomes, it makes it difficult to control potential confounding factors such as post-operative care, discharge criteria and robotic platform used, all of which could potentially lead to observation bias. However, considering all surgeons participated in the EARCS training programme, most surgeons applied relatively similar surgical techniques, therefore reducing surgical variability and operation-dependent observation bias. Finally, although data were collected from prospectively collated databases, the study was retrospective in nature and as a result there was no power calculation. However, this offers the advantage of the data being contemporary in nature—“real world” data—rather than data collected as part of a surgical trial that possibly includes an element of performance bias [42, 43]. In addition, all consecutive cases were included which minimises selection bias.

In conclusion, this study suggests that colorectal surgeons learning how to perform robotic surgery under the EARCS-structured training pathway can safely achieve short-term surgical outcomes comparable to their trainers and overcome the learning process in a way that minimises patient harm while achieving competency. Future studies including functional and long-term oncological data would be of great value in order to illuminate a more holistic comparison and strengthen the conclusions of this study.

## Supplementary Information

Below is the link to the electronic supplementary material.

(PDF 464 kb)
