# Supplementary material for: Short-term clinical outcomes of a European training programme for robotic colorectal surgery
Source: Surg Endosc. 2020 Dec 7;35(12):6796–806. doi: 10.1007/s00464-020-08184-1 (PMC8599412; doi:10.1007/s00464-020-08184-1)
Supplement: Supplementary file 1 — (PDF 464 kb) [file 464_2020_8184_MOESM1_ESM.pdf]

EARCS

European Academy of Robotic Colorectal Surgery

TABLE OF CONTENTS

PAGE

TABLE OF CONTENTS I

1 KEY ROLES AND CONTACT INFORMATION 1

2 INTRODUCTION 4

2.1 Aim 4

2.2 Background and Rationale 4

3 CURRICULUM 7

3.1 Selection Criteria for Candidate Trainee Surgeons 7

3.2 Steps of Training 7

3.3 Final Assessment for Competence 7

3.4 Timeline 8

3.5 Funding 8

1 KEY ROLES AND CONTACT INFORMATION

**EARCS Directors:**	Prof. Amjad Parvaiz, MBBS, FRCS, FRCS (Gen & Colorectal Surgery).<br><br>Professor of Surgery<br>National Trainer in Laparoscopic Colorectal Surgery<br>Director of EARCS<br><br>Poole Hospital, UK<br>University of Portsmouth, UK<br>Champalimaud Foundation, Portugal<br><br>Champalimaud Centre for the Unknown<br>Avenida da Brasilia<br>Lisbon, Portugal 1400-038<br>Tel: (+351) 210 480 193 – ext. 4666<br>Fax: (+351) 210 496 190<br>Email: apcheema@yahoo.com
	Prof. Giuseppe Spinoglio<br><br>Director of EARCS<br><br>Consultant Surgeon<br>Casa di Cura San Pio X, Milan, Italy<br><br>Professor of Robotic Surgery<br>Amedeo Avogadro University, Novara, Italy<br><br>Studio Medico Venezia 37<br>Per il Dr Giuseppe Spinoglio<br>Via Venezia 37<br>15121 Alessandria Italy<br>Mob: +39 335 6063411<br>Email: giuseppe.spinoglio90@gmail.com
**Administrative Coordinator:**	Ms. Rachelle Bissett-Amess<br>Coordinator<br>European Academy of Robotic Colorectal Surgery (EARCS)<br>Champalimaud Centre for the Unknown<br>Avenida da Brasilia, Lisbon, Portugal 1400-038<br>Tel: (+351) 210 480 193 – ext. 4666<br>Mob: (+351) 962 752 530<br>Fax: (+351) 210 496 190<br>Email: earcs@fundacaochampalimaud.pt

2 INTRODUCTION

The European Academy of Robotic Colorectal Surgery (EARCS) is a robotic surgical training programme designed to improve patient outcomes for colorectal cancer patients by improving the entire surgical learning pathway for robotic (minimally invasive) surgery: training, mentor relationships and data evaluation. The Academy was developed to ensure that robotic colorectal surgical training is conducted under safe and controlled conditions in order to manage the risks to patients when introducing a new surgical technique.

EARCS is directed by some of the eminent European experts in the field, some of whom have experience in the UK National Training Programme (NTP) for Laparoscopic Colorectal Surgery (LAPCO). Training is given by EARCS faculty on robotic surgical systems in the UK, Europe and some other international sites. To date, the Academy trains surgeons on the use of the daVinci surgical robot which is widely available for use, but is open to training with other systems as they become commercially available. Training is given to surgeons at sites interested in adopting robotic colorectal surgery and who have agreed to participate.

2.1 Aim

The aim of the Academy is to create a reproducible, repeatable training structure for new colorectal surgeons by:

1. Training experienced colorectal surgeons to perform robotic colon and rectal surgeries, so they can become proficient and independent in performance of such surgeries.

2. Receiving accreditation to key societies on the training pathway and approach.

3. Collecting surgical training assessment data to analyse trainee's performance during and after training (learning curve studies).

4. Collecting clinical outcomes data to analyse the effectiveness of training (oncological outcome studies).

2.2 Background and Rationale

Laparoscopy has become the gold standard for the treatment of colorectal cancer in the Western World. It is associated with better short-term outcomes, like less postoperative pain, less blood loss, reduced analgesia requirement and shorter hospital stay.

Whilst Laparoscopic colonic surgery is relatively easy to perform with no impact on oncological outcomes, Laparoscopic rectal cancer surgery is associated with higher morbidity and higher rates of conversion to open procedure.

Pelvic surgery for rectal cancer presents the technical challenges of bony confines, access and exposure issues, that makes this surgery technically very challenging. With the robotic system, some of these technical issues of exposure, access and wrist manipulation can be overcome with appropriate training.

There is a growing interest in the adoption of robotic surgery particularly for rectal resection. With increased popularity, there is real need for a professional training body to provide training structure to meet this demand and for clinical evidence to determine whether patient outcomes are improved.

With this in mind, a Consensus meeting for the creation of such a body took place at the Champalimaud Foundation Lisbon, Portugal on 20 June 2014. The meeting was co-chaired by Prof. Bill Heald and Prof. Amjad Parvaiz with the participants listed in **Table 1**.

Table 1. List of Consensus Meeting Attendees

Title	First Name	Last Name	Institution	City	Country
Prof.	Sergio	Alfieri	Università Cattolica del Sacro Cuore	Rome	Italy
Prof.	Thomas	Becker	Universitätsklinikum Schleswig-Holstein	Kiel	Germany
Dr.	Jerzy	Draus	Lanssjukhuset	Halmstad	Sweden
Prof.	Jan	Egberts	Universitätsklinikum Schleswig-Holstein	Kiel	Germany
Dr.	Nuno	Figuereido	Champalimaud Foundation	Lisbon	Portugal
Dr.	Marcos	Gomez Ruiz	Hospital Universitario de Marqués de Valdecilla	Santander	Spain
Dr.	Oliver	Haase	Charité Universitätsmedizin	Berlin	Germany
Prof.	Bill	Heald	Champalimaud Foundation	Lisbon	Portugal
Dr.	Henrik	Iversen	Karolinska Sjukhus	Stockholm	Sweden
Prof.	Martin	Kreis	Charité Universitätsmedizin	Berlin	Germany
Dr.	Henrik	Loft Jakobsen	Herlev Hospital	Herlev	Denmark
Dr.	Benno	Mann	Augusta Kranken Anstalt gGmbH	Bochum	Germany
Prof.	Danilo	Miskovic	University of Leeds	Leeds	UK
Prof.	Amjad	Parvaiz	Queen Alexandra Hospital	Portsmouth	UK
Prof.	Tero	Rautio	Oulu University Hospital	Oulu	Finland
Prof.	Philippe	Rouanet	CRLC Val d'Aurelle	Montpellier	France
Prof.	Giuseppe	Spinoglio	Istituto Clinico Humanitas	Milan	Italy
Dr.	Niels	Thomassen	Aarhus Universitetshospital	Aarhus	Denmark
Dr.	Alain	Valverde	Hospitalier Diaconesses Croix Saint-Simon	Paris	France

Following useful discussions around the structure of such a body and the curriculum for training, consensus agreement was reached to provide such a resource for European colorectal surgeons. The European Academy of Robotic Colorectal Surgery (EARCS) was founded on 20 June 2014 with Prof. Bill Heald as Honorary Chairperson, Prof. Amjad Parvaiz and Prof. Giuseppe Spinoglio as the founding Directors and the training faculty listed in **Table 2** (updated to include new Faculty accepted to the Academy). Faculty members contribute as trainers (proctors), on the surgical competence assessment committee and/or on the educational committee.

Table 2. Current List of Faculty

Title	First Name	Last Name	Institution	City	Country
Prof.	Sergio	Alfieri	Università Cattolica del Sacro Cuore	Rome	Italy
Mr.	Schwan	Amin	Sheffield Teaching Hospital	Sheffield	UK
Dr.	Heiko	Aselmann	Universitätsklinikum Schleswig-Holstein	Kiel	Germany
Prof.	Thomas	Becker	Universitätsklinikum Schleswig-Holstein	Kiel	Germany
Dr.	Paolo Pietro	Bianchi	International School of Robotic Surgery - Ospedale Misericordia	Grosseto	Italy
Dr.	Rogier	Crolla	Amphia Ziekenhuis	Breda	Netherlands
Dr.	Nicola	De'Angelis	Henri Mondor Hospital – University of Paris Est, Créteil (UPEC)	Paris	Italy
Prof.	Jan	Egberts	Universitätsklinikum Schleswig-Holstein	Kiel	Germany
Mr.	Golam	Farook	Sunderland Royal Hospital	Sunderland	UK
Dr.	Nuno	Figuereido	Champalimaud Foundation	Lisbon	Portugal

Title	First Name	Last Name	Institution	City	Country
Dr.	Roger	Gerjy	Danderyds University Hospital	Stockholm	Sweden
Dr.	Marcos	Gomez Ruiz	Hospital Universitario de Marqués de Valdecilla	Santander	Spain
Dr.	Henrik	Loft Jakobsen	Herlev Hospital	Herlev	Denmark
Prof.	David	Jayne	St James's University Hospital	Leeds	UK
Dr.	Ahyan	Kuzu*	University of Ankara - İbn-i Sina Hospital	Anakara	Turkey
Dr.	Fabrizio	Luca	Loma Linda University Health	Loma Linda	US
Dr.	Benno	Mann	Augusta Kranken Anstalt gGmbH	Bochum	Germany
Prof.	Danilo	Miskovic	University of Leeds	Leeds	UK
Prof.	Amjad	Parvaiz	Queen Alexandra Hospital	Portsmouth	UK
Dr.	Daniel	Perez	Universitätsklinikum Hamburg-Eppendorf	Hamburg	Germany
Dr.	Fabio	Priora	SS. Antonio e Biagio and C. Arrigo Hospitals	Alessandria	Italy
Dr.	Tasheen	Qureshi	Poole Hospital NHS Foundation Trust	Poole	UK
Prof.	Tero	Rautio	Oulu University Hospital	Oulu	Finland
Prof.	Philippe	Rouanet	CRLC Val d'Aurelle	Montpellier	France
Dr.	Irshad	Shaikh	Norfolk and Norwich University Hospital	Norwich	UK
Dr.	Matej	Skrovina	Hospital and Oncology Centre Novy Jicin	Novy Jicin	Czech Republic
Prof.	Giuseppe	Spinoglio	Istituto Clinico Humanitas	Milan	Italy
Dr.	Niels	Thomassen	Aarhus Universitetshospital	Aarhus	Denmark
Dr.	Alain	Valverde	Hospitalier Diaconesses Croix Saint-Simon	Paris	France
Dr.	George	Van der Schelling	Amphia Ziekenhuis	Breda	Netherlands
Dr.	Gintautas	Virakas	Augusta Klinika Bocham Hattigen	Bocham	Germany

*Anatamy expert only.

3 CURRICULUM

3.1 Selection Criteria for Candidate Trainee Surgeons

Experienced colorectal surgeons who wish to enter the EARCS training programme need to apply to the EACRS coordinating office for review and approval. The following selection criteria apply:

- Consultant colorectal surgeon with volume of colorectal practice
- Support from Hospital executives
- Access to Robotic system on regular basis
- Experience in Laparoscopic surgery desirable but not essential.

3.2 Steps of Training

Clinical training normally takes place through a combination of both "case-observation" at the hospital of the EARCS Faculty Member (proctor), and "proctored training sessions". Proctored training sessions involve the EARCS Faculty Member attending the trainee surgeon's hospital where the trainee performs a case under direct supervision of the EARCS Faculty Member. Trainee surgeons are expected to pass an assessment to progress to the next step of training. The steps of training include:

- **Case Observations at Faculty Member Institutions:**

  This step includes attending lectures, which cover thorough knowledge of pelvic anatomy and the theory behind Total Mesorectal Excision (TME) surgery. This two-day course will involve lecture-based teaching as well as live case observation.

- **System and Dissection Training Course (two consecutive days):**

  Day One: Console/System Training Course - This course will be delivered at a designated training centre to provide insight and competence with the system and console for the trainees. There is a process of assessment at the end of the training. This is designed to introduce trainees to the concept of applying the robotic system to perform surgical procedures. The porcine training model is used to achieve this.

  Day Two: Procedure Specific Cadaveric Course - This course is aimed at delivering hands on training using cadaveric material at a designated training centre. The course will be delivered under the direct supervision of EARCS faculty.

- **Hands-On Training at the Faculty Members Hospital and Trainees Own Hospital:**

  The clinical hands-on training consists of two proposed models. Firstly, the trainees are encouraged to go to the institution of one of the EARCS faculty (of their choice) to participate in hands-on training. This is followed by faculty visits to the trainee's hospital to supervise training in that setting. Trainees are expected to do approximately five left sided (sigmoid colon) resections and five rectal resections under the direct supervision of the faculty (proctor). The number of required proctored cases varies from trainee to trainee and trainees can only progress to the final assessment phase upon approval of the proctor.

3.3 Final Assessment for Competence

Clinical training is assessed and scored using Global Assessment Score (GAS) forms, completed by the trainee and trainer/proctor and submitted to the Coordinating Centre (GAS form attached). At the end of the total number of training cases performed, trainees are expected to submit two videos of self-performed robotic colon and rectal resection for 'blinded' assessment in order to exit the programme. The videos will be blindly assessed by two EARCS faculty members using Robotic Colorectal Assessment (RCAT) forms (RCAT form

attached). If the assessors feel that the trainee is competent in performing a robotic colon and rectal surgery alone, they will recommend solo practice and EARCS will issue a certificate of competence. If the assessors feel that the trainee has not yet reached a competent level, they will recommend further supervised practice before resubmitting.

3.4 Timeline

The timeline for all of the steps to be completed is expected to be around 16 weeks. It is expected that clear demonstration of competence will be achieved before moving onto the next phase of training.

3.5 Funding

Funding for central coordination of EARCS and proctoring was supported by Intuitive Surgical (2015-2019) and the Champalimaud Foundation (2020). Funding to attend training will be the responsibility of the trainees. All data collected is solely owned by EARCS.

EARCS
European Academy of
Robotic Colorectal Surgery

Global Assessment Score (GAS) Form
Robotic Colorectal Surgery

A. CASE INFORMATION

**Form completed by:**	
**Date of surgery (dd/mm/yyyy):**	
**Name of trainee:**	
**Name of proctor:**	
**Name of procedure (refer to back):**	

B. GLOBAL ASSESSMENT SCORE (GAS)

1 – Step done by trainer/ other trainee/ not required in this procedure
2 – Physical input by trainer required (partly performed by trainer)
3 – Substantial verbal input by trainer required
4 – Only minor verbal input by trainer required
5 – Independent and competent performance, no substantial trainer input
6 – Masterful performance, couldn't be better
NA – Not Applicable

**ROBOT DOCKING**							
Laparoscopic access	1 ○	2 ○	3 ○	4 ○	5 ○	6 ○	NA ○
Robot positioning	1 ○	2 ○	3 ○	4 ○	5 ○	6 ○	NA ○
Docking of robotic arms	1 ○	2 ○	3 ○	4 ○	5 ○	6 ○	NA ○
**COLONIC DISSECTION**							
Transection of vascular pedicle	1 ○	2 ○	3 ○	4 ○	5 ○	6 ○	NA ○
Mobilisation of colon (medial and lateral)	1 ○	2 ○	3 ○	4 ○	5 ○	6 ○	NA ○
Splenic flexure mobilization	1 ○	2 ○	3 ○	4 ○	5 ○	6 ○	NA ○
**TOTAL MESORECTAL EXCISION (TME)**							
Posterior plane and upper right and left lateral	1 ○	2 ○	3 ○	4 ○	5 ○	6 ○	NA ○
Anterior plane	1 ○	2 ○	3 ○	4 ○	5 ○	6 ○	NA ○
Lateral dissection	1 ○	2 ○	3 ○	4 ○	5 ○	6 ○	NA ○
Low pelvic dissection	1 ○	2 ○	3 ○	4 ○	5 ○	6 ○	NA ○
Transection of rectum	1 ○	2 ○	3 ○	4 ○	5 ○	6 ○	NA ○
**RESECTION AND ANASTOMOSIS**							
Exteriorisation/ resection of specimen	1 ○	2 ○	3 ○	4 ○	5 ○	6 ○	NA ○
Anastomosis	1 ○	2 ○	3 ○	4 ○	5 ○	6 ○	NA ○

Notes:

EARCS
European Academy of Robotic Colorectal Surgery

Global Assessment Score (GAS) Form

Robotic Colorectal Surgery

NAME OF PROCEDURE

Right hemicolectomy
Extended right hemicolectomy
Transverse colectomy
Left hemicolectomy
Sigmoid colectomy
Anterior resection TME
Anterior resection PME
Abdominoperineal resection APR
Abdominoperineal resection APER
Hartmann's procedure
Panproctocolectomy
Other (please name)

INTERIM Robotic Colorectal Video Assessment Form

Robotic Low Anterior Resection

A. CASE INFORMATION

**Surgeon ID:**	
**Video ID:**	
**Assessment form complete by:**	
**Procedure name:**	

B. ASSESSMENT

Please watch the case video and make a subjective assessment on the competency of the subject surgeon as follows:

1. Assess the following major tasks for competency.
2. Make a final recommendation of either "Competent" or "Resubmit"
3. Justify your final recommendation.

Note: for the surgeon to pass this final sign-off phase, he/she must receive a final recommendation of "Competent". A final recommendation of "Resubmit" means that the surgeon will be invited to resubmit his/her videos for final assessment once he/she has completed further practice, which could include advanced proctoring.

1. EXPOSURE AND VASCULAR LIGATION

2. MOBILISATION INCLUDING THE SPLENIC FLEXURE

3. PROCTECTOMY

3.1. POSTERIOR PLANE DISSECTION

3.2. ANTERIOR PLANE DISSECTION

3.3. LATERAL PLANE DISSECTION

4. TRANSECTION

5. ANASTOMOSIS

C. FINAL RECOMMENDATION

**Competent or Resubmit:**	

D. JUSTIFICATION
